# Clinical, Laboratory, Histological, Radiological, and Metabolic Features and Prognosis of Malignant Pleural Mesothelioma

**DOI:** 10.3390/medicina58121874

**Published:** 2022-12-19

**Authors:** Yuan Zhang, Ran Li, Yumei Gu, Yuerong LiZhu, Xiaofang Liu, Shu Zhang

**Affiliations:** 1 Department of Respiratory and Critical Care Medicine, Beijing Institute of Respiratory Medicine and Beijing Chao-Yang Hospital, Capital Medical University, Beijing 100020, China; 2 Department of Respiratory and Critical Medicine, Beijing Tongren Hospital, Capital Medical University, Beijing 100730, China; 3 Department of Pathology, Beijing Chao-Yang Hospital, Capital Medical University, Beijing 100069, China

**Keywords:** malignant pleural mesothelioma, histological features, radiological characteristics, PET-CT metabolic characteristics, prognosis

## Abstract

*Background:* Malignant pleural mesothelioma (MPM) is an aggressive and rare malignant pleural tumor. *Methods:* MPM patients diagnosed in Beijing Chaoyang Hospital and Beijing Tongren Hospital were the focus of this study. We collected and analyzed the histological, radiological, and metabolic features of MPM patients. At the same time, Cox univariable and multivariable analyses were used to explore the laboratory risk factors affecting the prognosis of MPM patients. *Results:* A total of 129 MPM patients were included in this study. MPM includes three main histological subtypes: epithelioid, sarcomatoid and biphasic. Among them, epithelial subtypes accounted for the highest proportion. Calretinin, Wilms’ tumor gene (WT1), cytokeratin 5/6 (CK5/6), and D2-40 were the most useful mesothelial markers to support a MPM diagnosis. The imaging features of MPM patients are pleural thickening and pleural effusion. In PET-CT, the affected pleura showed obvious high uptake of tracer, and the degree was related to the specific subtype. The median follow-up time was 55.0 (30.0, 94.0) months. A total of 92 (71.3%) patients died during follow-up. The median survival time of patients was 21.0 (9.0, 48.0) months. The Cox multivariable analysis showed that age [hazard ratio (HR), 1.824; 95% confidence interval (CI) 1.159–2.872; *p* = 0.009; uncorrected], ESR (HR, 2.197; 95% CI 1.318–3.664; *p* = 0.003; with Bonferroni correction), lymphocytes (HR, 0.436; 95% CI 0.258–0.737; *p* = 0.002; with Bonferroni correction), platelets (HR, 1.802; 95% CI 1.084–2.997; *p* = 0.023; uncorrected) and total protein (HR, 0.625; 95% CI 0.394–0.990; *p* = 0.045; uncorrected) were independent risk factors for prognosis, after adjusting for confounding factors. *Conclusions:* Age, ESR, lymphocytes, platelets and total protein may be related to the prognosis of MPM patients. Summarizing the histological, radiological, and metabolic features of MPM patients in the two centers can increase clinicians’ understanding of this rare tumor.

## 1. Introduction

Malignant pleural mesothelioma (MPM) is a rare invasive tumor originating from pleural mesothelial cells and mainly associated with asbestos exposure [1,2]. In the recent GLOBOCAN study, MPM accounted for 0.3% of total cancer deaths [3]. It is expected that the incidence rate and mortality rate will continue to increase worldwide [4,5]. Due to its aggressive characteristics, the 5-year survival rate of patients is less than 5% [6]. The median survival time range was 12–36 months [7,8].

MPM mainly includes the epithelial subtype, sarcomatoid subtype and biphasic subtype [9]. Because of its unique histological characteristics, it has strong heterogeneity in its clinical, laboratory, histological, radiological, metabolic features and prognosis [10,11]. At present, the description of its characteristics still needed to be further improved.

Determining the prognostic risk factors of MPM will help patients to obtain more reasonable treatment and more clinical benefits [12]. Peripheral blood is sensitive and easy to obtain. For clinicians, it is necessary to pay attention to the routine reexamination of patients’ tumor status. Previous studies showed that some of the indicators that can reflect the systemic inflammatory status of patients, such as the neutrophil-to-lymphocyte ratio (NLR) [13,14] platelet-to-lymphocyte ratio (PLR) [15] and lymphocyte-to-monocyte ratio(LMR) [16], significantly correlate with the prognosis of MPM patients [17].

The purpose of this study was to collect data on patients diagnosed with MPM in two centers in China; summarize the clinical, imaging, nuclear medicine and histological characteristics of MPM patients; and explore the independent risk factors affecting the prognoses of patients.

## 2. Methods

### 2.1. Study Population

This malignant pleural mesothelioma cohort included patients diagnosed and treated in Beijing Chaoyang Hospital and Beijing Tongren Hospital from January 2010 to May 2022. Patients who met the following inclusion criteria were included in a retrospective observation cohort: pathologically proven malignant pleural mesothelioma, aged over 18 years. The exclusion criteria were as follows: incomplete clinical, laboratory and follow-up information. All the procedures were performed in line with the principles of the Declaration of Helsinki. This study was approved by the Ethics Committee of Beijing Chaoyang Hospital.

### 2.2. Clinical and Laboratory Data Collection

Patient-related factors (age, sex, smoking history, asbestos exposure history, Eastern Cooperative Oncology Group Performance Status (ECOG PS)), tumor-related factors (tumor diagnosis mode, histological subtype, and anti-tumor treatment including surgery, chemotherapy and anti-angiogenesis therapy) and laboratory variables at the time of diagnosis (erythrocyte sedimentation rate (ESR), sodium, potassium, calcium, chloride, white blood cell (WBC), neutrophil, lymphocyte, hemoglobin, platelet, total protein, albumin, glucose, creatinine, total bilirubin) were obtained from electronic medical records.

### 2.3. Diagnostic Procedures

The specific method of diagnosis was determined by the respiratory physician, interventional physician, and thoracic surgeon after careful discussion.

Some patients were poor surgical candidates, and in these cases, pleural effusion cytology was used to confirm the diagnosis.

Percutaneous pleural needle biopsies were usually performed with a Supercore biopsy instrument 18 ga × 15 cm used with an optional co-axial needle MCXS1815LX (Argon Medical, Texas, TX, USA) or Supercore biopsy instrument 18 ga × 9 cm used with an optional co-axial needle MCXS1809LX (Argon Medical, Texas, TX, USA).

The medical thoracoscopic procedures were completed by an experienced respiratory intervention physician. The patient was positioned in a lateral position with the affected side facing upward. After local anesthesia, a disposable trocar was inserted, and a semi-rigid pleuroscope (Olympus LTF-240, Tokyo, Japan) was used to explore the thoracic changes in detail. Biopsies were taken from multiple sites for pathological examination. Finally, a thoracic drainage tube was inserted for drainage.

All the patients received 2-port unilateral video-assisted thoracoscopic surgery (VATS) with double lumen endotracheal tube and one-lung ventilation under general anesthesia, which was performed by a committee-certified thoracic surgeon. After the pathology was obtained, a drainage tube for pleural effusion was placed in the chest cavity.

### 2.4. Histology and Immunohistochemical Staining

Hematoxylin and eosin (H&E) and immunohistochemical staining were performed using the Ventana HE 600 automated staining system (Ventana Medical Systems, Inc., Tucson, AZ, USA) and the Ventana Bench Mark ULTRA Autostainer (Ventana Medical Systems, Inc., Tucson, AZ, USA). H&E and immunohistochemical staining were reviewed by two mid-career pathologists who were blinded to clinical information.

### 2.5. CT Technique and Image Analysis

Scanning was performed in the supine position of the patient. The scan range was from the chest entrance to the level of the diaphragm. At the beginning of the scan, the patient is required to hold their breath after inhalation. The scanning equipment was a third-generation, dual-source computer tomography (CT) (SOMATOM Definition Force; Siemens Healthcare, Forchheim, Germany). All CT images were reconstructed to a layer thickness of 5.0 mm. The GE picture archiving and communication system was used for film reading. Two experienced radiologists who were blinded to clinical information reviewed the images, respectively.

### 2.6. ^18^F-FDG PET-CT Technique and Image Analysis

All patients received intravenous injections of [18] F-FDG according to body weight. After 60 min of injection, scanning was performed with a high spatial resolution, full-ring PET scanner (Biograph MCT, Siemens Healthcare, Erlangen, Germany). AW Volume Share 2 software was used to reconstruct imaging data. PET/CT datasets were reviewed and analyzed by two experienced nuclear medicine physicians and PET/CT experts who were blind to clinical information

### 2.7. Clinical Outcome Evaluation

The main outcome of this study was the prognosis of the patients. OS was determined as the time from diagnosis to death or the end of follow-up. The outcome events of the patients were determined through the electronic medical record system and telephone follow-up. The last telephone follow-up was in August 2022. MPM patients are routinely examined at least once every half year within 2–3 years after diagnosis, and at least once every year thereafter.

### 2.8. Statistical Analysis

Continuous variables were described by using the median and interquartile range (IQR). Receiver operated characteristics (ROC) curves were constructed to determine cutoff values of laboratory indicators that yield the joint maximum sensitivity and specificity [18,19,20]. Continuous variables are converted to category variables by optimal cut-off values. Categorical variables were described as frequencies and percentages. The survival curve was drawn by Kaplan–Meier, and the difference between the two groups was tested by the log-rank test. Univariable and multivariable analyses were analyzed by Cox regression analysis. In COX multivariable analysis, age, sex, histological subtype and treatment are used for correction, and also included in the indicators that are generally concerned by clinical researchers and have statistical significance in univariable analysis. The threshold was defined as a Bonferroni correction of *p* < 0.05 (*p* < 0.05/10 = 0.005). Further, we also performed an exploratory analysis (the threshold was set as *p* < 0.05), which reflects the exploratory nature. All the analyses were performed using the SPSS software (version 25.0; IBM, Armonk, NY, USA). Professional epidemiologists reviewed the statistical methods in this paper.

## 3. Results

### 3.1. Patient Characteristics

A total of 129 patients with MPM diagnosed from Beijing Chaoyang Hospital and Beijing Tongren Hospital were included in this cohort. The median age at diagnosis was 65.5 years. Forty-five percent (58/129) of the patients were female. A total of 59.7% (77/129) of the patients never smoked. A total of 20.9% (27/129) of the patients had a clear history of asbestos exposure. A total of 82.9% (107/129) patients had an ECOG PS of 0–1. The tumor characteristics of the cohort were also recorded. For diagnosis, 3 (2.3%) patients had pleural effusion aspirated for cellular wax block, 19 (14.7%) patients had an ultrasound-guided pleural biopsy, 12 (9.3%) patients were diagnosed with VATS, and 95 (73.6%) patients underwent medical thoracoscopy. In total, 82 patients (63.6%) were pathologically identified as epithelial subtypes; there were 47 patients (36.4%) with non-epithelioid subtypes including sarcomatoid, biphasic, and unclassified subtypes. It should be noted that the unclassified subtypes are usually difficult to classify the intrinsic specific subtypes due to the small sample size. Most patients received chemotherapy alone or in combination with anti-angiogenic therapy (83.7%; *n* = 108). A total of 68 (52.7%) patients were found to have received anti-angiogenic therapy (Table 1). The results of laboratory variables at the time of diagnosis are also detailed in Table 1.

### 3.2. Cut-off Values for Laboratory Variables and Survival Analysis

According to the ROC curve, we calculated the cut-off value of all laboratory parameters, such as the cut-off value of ESR of 20.5. All patients were divided into two groups according to the cut-off value. There were 75 patients (58.1%) in the low-concentration group (ESR ≤ 20.5) and 54 patients (41.9%) in the high-concentration group (ESR > 20.5). Other laboratory indicators were calculated in the same way (Table 2). The median follow-up time was 55.0 (30.0, 94.0) months. A total of 92 (71.3%) patients died during follow-up. The median survival time of patients was 21.0 (9.0, 48.0) months.

The Cox univariable analysis showed that treatment (*p* = 0.012), ESR (*p* < 0.001), sodium (*p* = 0.017), potassium (*p* = 0.023), lymphocyte (*p* = 0.009), platelet (*p* = 0.008), and total protein (*p* = 0.043) might be associated with prognosis. Other indicators were not associated with prognosis (Table 2). After adjustment for age, sex, histological subtype and treatment, the Cox multivariable analysis showed that age [hazard ratio (HR), 1.824; 95% confidence interval (CI) 1.159–2.872; *p* = 0.009; uncorrected], ESR (HR, 2.197; 95% CI 1.318–3.664; *p* = 0.003; with Bonferroni correction), lymphocyte (HR, 0.436; 95% CI 0.258–0.737; *p* = 0.002; with Bonferroni correction), platelet (HR, 1.802; 95% CI 1.084–2.997; *p* = 0.023; uncorrected) and total protein (HR, 0.625; 95% CI 0.394–0.990; *p* = 0.045; uncorrected) were independent risk factors for prognosis (Table 3).

**Figure 1:**
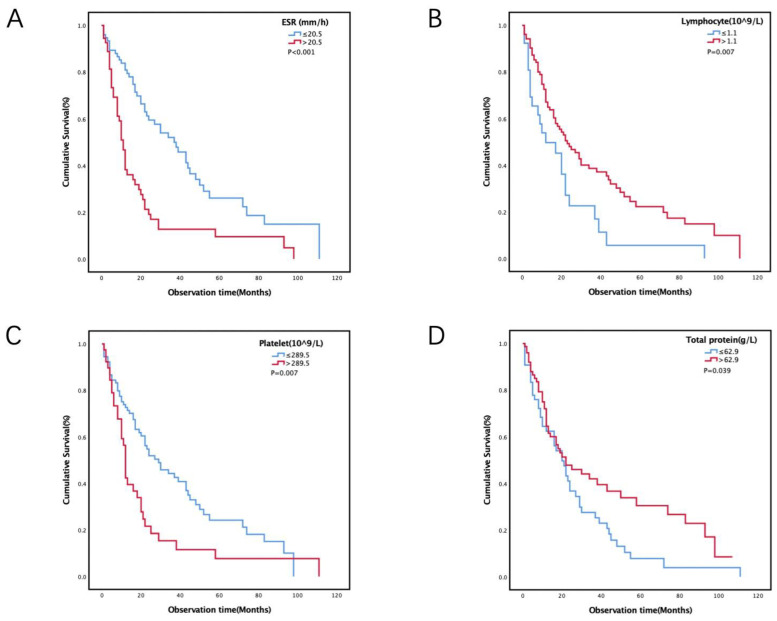
Association between laboratory variables and overall survival in patients with malignant pleural mesothelioma. (**A**) Kaplan–Meier curve of overall survival stratified by erythrocyte sedimentation rate (*p* < 0.001). (**B**) Kaplan–Meier curve of overall survival stratified by lymphocyte (*p* = 0.007). (**C**) Kaplan-Meier curve of overall survival stratified by platelet (*p* = 0.007). (**D**) Kaplan–Meier curve of overall survival stratified by total protein (*p* = 0.039).

Patients in the low-ESR group and the low-platelet group had a better prognosis than those in the high-ESR group (Figure 1A) and high-platelet group (Figure 1C). Compared with the low-lymphocyte group and low-total protein group, the high-lymphocyte group (Figure 1B) and high-total protein group (Figure 1D) had a better prognosis.

### 3.3. Thoracoscopic Findings and Pathological Features

Due to the difference in samples, different kinds of immunohistochemical staining were performed on different patients. Therefore, we summarized that the sample size included in the immunohistochemistry table is different. We found that in MPM, most of the Calretinin, CK5/6, D2-40, MA, WT-1, CK7, EMA, CK, Glut1, Vimentin and P53 were positive. At the same time, the proportion of negative expression of TTF-1, CEA, Desmin and Napsin-A was higher (Table 4).

**Figure 2:**
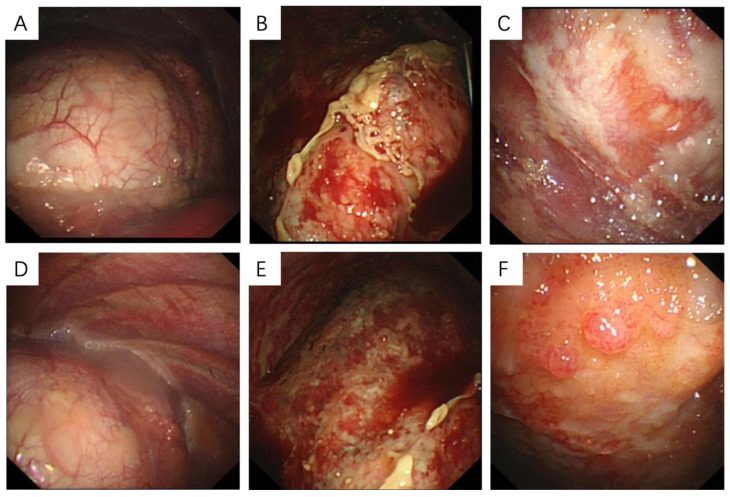
Thoracoscopy images in malignant pleural mesothelioma patients. (**A**,**D**) Epithelioid: There are a few white nodules on the parietal pleura and diaphragmatic pleura, accompanied by a small amount of pleural effusion. (**B**,**E**) Sarcomatoid: There are a large number of tough patchy masses on the parietal, visceral and diaphragmatic pleura, accompanied by a large number of bloody pleural effusion. (**C**,**F**) Biphasic: There are scattered white plaque-like lesions in the parietal layer and diaphragmatic pleura, accompanied by a large amount of yellow pleural effusion.

**Figure 3:**
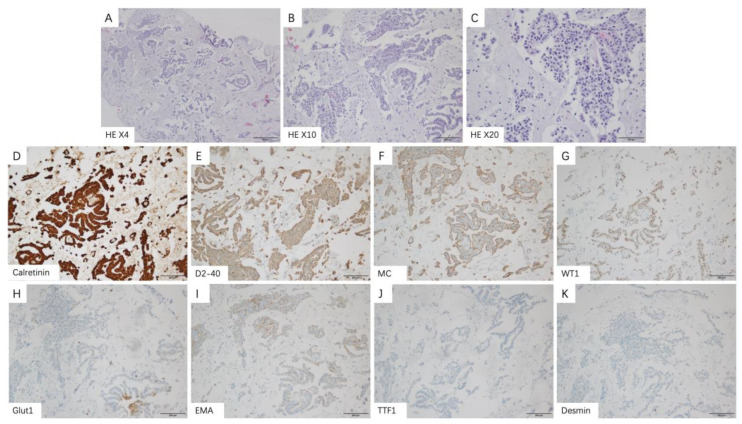
Representative histologic images and immunophenotype of epithelioid subtypes in malignant pleural mesothelioma patients. (**A**–**C**) Epithelioid subtypes show papillary arrangement and infiltrative growth of tumor cells (original magnification ×4 (**A**), ×10 (**B**) and ×20 (**C**)). The positive immune-expression of Calretinin (**D**), D2-40 (**E**), MC (**F**), WT1 (**G**). The partial positive immune-expression of Glut1 (**H**), EMA (**I**). The negative immune-expression of TTF1 (**J**), Desmin (**K**).

Medical thoracoscopy is a common invasive examination for MPM patients. Patients with epithelial subtypes can display the following characteristics during surgery: no obvious adhesion was found in the thoracic cavity; a small amount of pleural effusion can be observed in the chest cavity; smooth parietal layer and diaphragmatic pleura without obvious hyperemia and edema but with scattered white nodules exist; smooth visceral pleura without nodules (Figure 2A,D). Microscopically, tumor cells with eosinophilic cytoplasm and atypia of nuclei were seen in papillary arrangement and infiltrative growth. Immunohistochemical staining showed: Calretinin, D2-40, MC, WT1 (+), EMA, GLUT1 (partial+), TTF1, Desmin (−) (Figure 3A–K).

**Figure 4:**
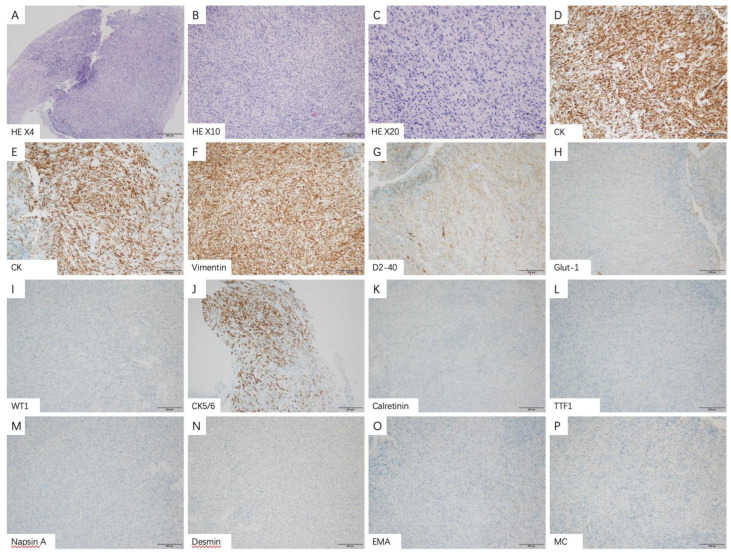
Representative histologic images and immunophenotype of sarcomatoid subtypes in malignant pleural mesothelioma patients. (**A**–**C**) Sarcomatoid subtypes show proliferation of pleural tissue and mesothelial cells (original magnification ×4 (**A**), ×10 (**B**) and ×20 (**C**)). The positive immune-expression of CK (**D**), CK7 (**E**), Vimentin (**F**). The partial positive immune-expression of D2-40 (**G**), Glut1 (**H**), WT1 (**I**), CK5/6 (**J**). The negative immune-expression of Calretinin (**K**), TTF1 (**L**), Napsin A (**M**), Desmin (**N**), EMA (**O**), MC (**P**).

In MPM patients with the sarcoma subtype, thoracoscopy showed that the parietal layer, visceral layer, diaphragm and pleura adhered to each other, and there was extensive hyperemia and thickening. Multiple patchy changes were found in the parietal layer, diaphragm and pleura, with partial fusion and tough texture. Multiple nodular changes can be seen on the pericardial surface. There is a large amount of bloody pleural effusion in the chest cavity (Figure 2B,E). Under the microscope, it could be seen that the pleural tissue and mesothelial cells proliferate, and the focal surface adheres to the exudation of cellulose. The stroma was infiltrated by lymphocytes and eosinophils, and short spindle cells with an increased nucleocytoplasmic ratio were seen. Immunohistochemical staining results showed that CK, CK7, Vimentin (+), D2-40, GLUT1, WT1, CK5/6 (partial+), Calretinin, TTF1, NapsinA, Desmin, EMA, MC (−) (Figure 4A–P).

**Figure 5:**
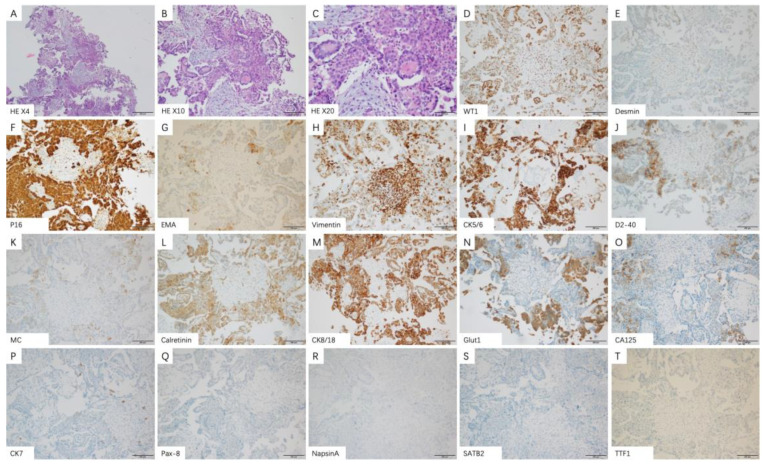
Representative histologic images and immunophenotype of biphasic subtypes in malignant pleural mesothelioma patients. (**A**–**C**) Biphasic subtypes show proliferation of pleural tissue and mesothelial cells (original magnification ×4 (**A**), ×10 (**B**) and ×20 (**C**)). The positive immune-expression of WT1 (**D**), Desmin (**E**), P16 (**F**), EMA (**G**), Vimentin (**H**), CK5/6 (**I**), D2-40 (**J**), MC (**K**), Calretinin (**L**), CK8/18 (**M**), Glut1 (**N**), CA125 (**O**). The negative immune-expression of CK7 (**P**), Pax-8 (**Q**), NapsinA (**R**), SATB-2 (**S**), TTF1 (**T**).

Under thoracoscopy, patients with the biphasic subtype showed a large number of yellow pleural effusions in the chest cavity. The parietal layer and diaphragmatic pleura were diffusely thickened, and white patchy lesions were diffusely distributed. The biopsy texture was very hard. Among them, there are scattered nodules of different sizes, and the texture of the nodules is relatively soft. No obvious nodules are found in the visceral pleura (Figure 2C,F). Microscopy showed that tumor cells infiltrated the tissue. Tumor cells are arranged in papillary, stratified, or solid patterns. The cells have remarkable heteromorphism. Mitotic images are easily seen. Spindle cells were scattered and interstitial fibrous tissue proliferated. Immunohistochemical staining results showed: WT1, Desmin, P16, EMA, Vimentin, CK5/6, D2-40, MC, Calretinin, CK8/18, Glut1, CA125 (+). CK7, Pax-8, NapsinA, SATB-2, TTF1 (−) (Figure 5A–T).

### 3.4. Radiological Features

**Figure 6:**
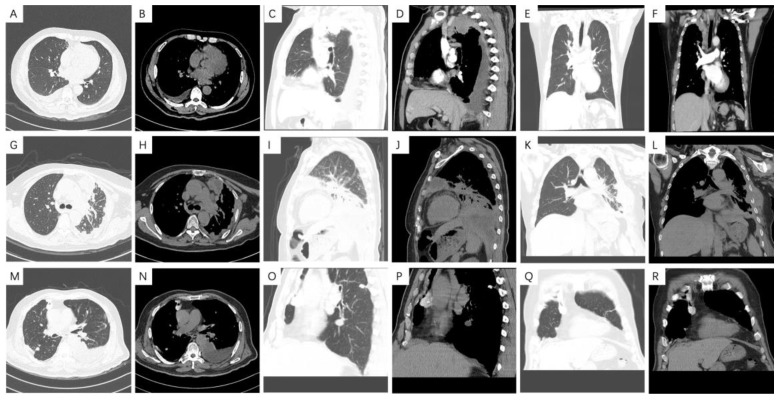
Computed tomography images in malignant pleural mesothelioma patients. (**A**–**F**) Epithelioid: A soft tissue mass shadow can be seen next to the right upper mediastinum, with a range of approximately 4.3 × 3.5 cm. The boundary between mass and mediastinum is not clear. The CT value of the plain scan was 51hu, and that of the enhanced scan was approximately 79hu. The pleura on the right side has multiple irregular thickening and obvious enhancement. There was a small amount of pleural effusion on the right side. An irregular nodular shadow can be seen in the right upper lung, with a size of approximately 1.6 × 1.5 cm. It is considered to be a malignant metastasis. (**G**–**L**) Sarcomatoid: The left pleura had uneven and obvious thickening. Soft tissue mass formation could be seen locally, and cystic changes could be seen in some lesions. Diffuse soft tissue density shadows could be seen on the left chest wall and abdominal wall, accumulating multiple ribs and thoracic vertebrae on the left side. The boundary between the lesion and pericardium, left ventricular wall, left pulmonary artery, thoracic aorta and left subclavian artery was unclear. (**M**–**R**) Biphasic: The broncho-vascular bundles of both lungs were thickened, with bronchial wall thickening, and some of them were stiff. The bilateral chest wall and pleura were extensively and unevenly thickened with multiple localized changes. Some of the tumors protruded into the lung field and some were accompanied by calcification. Multiple pleural effusions were found on both sides.

In the total of 129 patients, 20 patients had missing chest CT information. Therefore, we collected image data from only 109 patients. Among them, 63 patients (57.8%) had tumors in the left thoracic cavity and 39 patients (35.8%) had tumors in the right thoracic cavity. Seven (6.4%) lesions involved the bilateral thoracic cavity. A total of 36 (27.9%) cases involved interlobar pleura. The mediastinal pleura was involved in 15 (11.6%) patients. A total of 29 (26.6%) patients were found to have mediastinal lymph node enlargement. A total of 17 (15.6%) patients had reduced thoracic volume. A total of 2 (1.8%) patients had no pleural effusion, whilst 8 (7.3%) patients had a small amount of pleural effusion, 70 (64.2%) patients had moderate pleural effusion, and 29 (26.6%) patients had a large amount of pleural effusion. There was no obvious pleural thickening in 3 (2.8%) patients, regular pleural thickening in 20 (18.3%) patients, annular pleural thickening in 9 (8.3%) patients, and mass pleural thickening in 14 (12.8%) patients. A total of 63 (57.8%) patients had nodular pleural thickening. A total of 22 (20.2%) patients had pleural calcification. A total of 14 (12.8%) patients were found to have intrapulmonary metastasis. 2 (1.8%) patients were found to have rib metastasis. In total, 15 (13.8%) patients were found to have chest wall metastasis. No patient was found to have tumor necrosis and pericardial effusion (Figure 6A–Q). We found that there was no significant correlation between the above radiologic characteristics and histological subtypes (Table 5).

### 3.5. PET-CT Metabolic Characteristics

In this study, we found that only six patients received a PET-CT examination. The characteristics of PET-CT images of epithelial subtypes were as follows: focal increased uptake of tracer can be seen in the 6th–7th intercostal pleura on the right side, with a SUVmax of 2.5 and SUVmax of 2.6 on delayed imaging. In the right lung, a ground glass nodule shadow at the tip of the upper lobe, the cord shadow under the chest wall, and the pleural thickening and pleural effusion between the lobes can all be seen with increased tracer.

The characteristics of PET-CT images of sarcoma subtypes were as follows: multiple nodular thickening could be seen in the left pleura, and the tracer uptake was significantly increased with a SUVmax of 7.9. The boundary between nodules and pericardium was not clearly displayed. Increased tracer uptake could be seen in the left pleural effusion and the small lymph nodes of the hilar. A gas density shadow could be seen in the left thoracic cavity.

**Figure 7:**
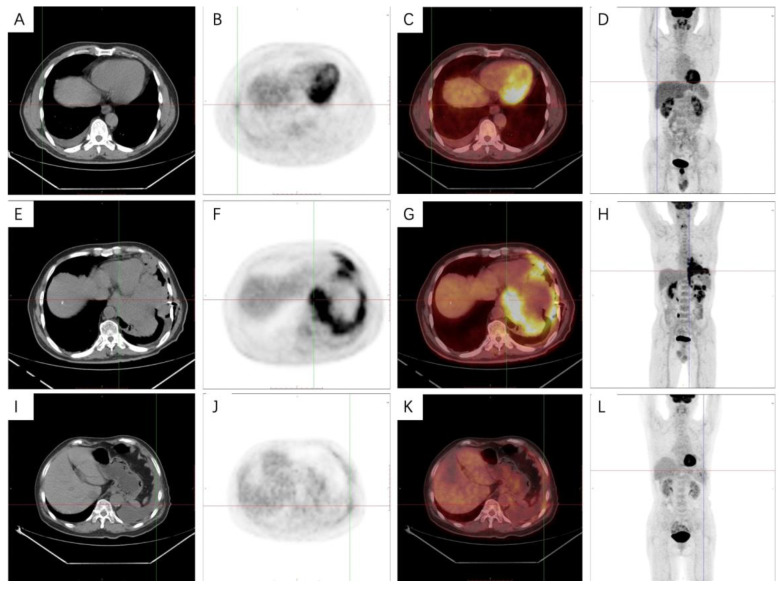
Positron emission tomography-computed tomography images in malignant pleural mesothelioma patients. (**A**–**D**) Epithelioid: Focal increased uptake of tracer can be seen in the 6th–7th intercostal pleura on the right side, with a SUVmax of 2.5 and SUVmax of 2.6 on delayed imaging. (**E**–**H**) Sarcomatoid: Multiple nodular thickening could be seen in the left pleura, and the tracer uptake was significantly increased with a SUVmax of 7.9. (**I**–**L**) Biphasic: Multiple thickening can be seen in the left pleura, with increased tracer uptake and a SUVmax of 3.3.

The characteristics of PET-CT images of biphasic subtypes are as follows: multiple thickening can be seen in the left pleura, with increased tracer uptake and a SUVmax of 3.3. The pleural thickening of the lingual segment of the upper lobe of the left lung was accompanied by a soft tissue density shadow under the pleura, which invaded the pericardium, accompanied by atelectasis of the lingual segment and increased tracer uptake (Figure 7A–L).

## 4. Discussion

Malignant mesothelioma is an aggressive tumor, which is increasing in frequency throughout the world. The prognosis is significantly heterogeneous. In this study, the clinical, laboratory, radiological, nuclear medicine, histological and other indicators of MPM patients in the two centers were comprehensively collected and analyzed. We summarize the unique radiological and histological features of MPM patients. The risk factors affecting prognosis were also analyzed.

### 4.1. Prognostic Risk Factors for Malignant Pleural Mesothelioma

The Cox multivariable analysis showed that age, ESR, lymphocytes, platelets and total protein might affect the prognosis of MPM patients. Compared with elderly patients, young patients have fewer underlying diseases. Moreover, young patients have better tolerance to adverse reactions that may be brought about by anti-tumor treatment. Therefore, the survival time may be longer. This is consistent with previous studies [21,22].

Previous studies have shown that in tumor patients, the chronic inflammatory state that persists for a long time will affect the production of cytokines and disrupt the immune balance between the immune system and tumor growth [23,24,25], thereby accelerating the progression of the tumor and leading to a poor prognosis [26,27].

The results of this study show that lower ESR, fewer platelets and more lymphocytes may indicate a better prognosis. These inflammatory indicators in peripheral blood at the time of diagnosis are readily available and may reflect the systemic inflammatory status of MPM patients. Elevated ESR levels have been shown to be associated with tumorigenesis and poor survival in a variety of malignancies, such as lung tumors, blood tumors, and colorectal tumors [28,29,30,31].

An increased platelet count is a negative prognostic factor for survival, which has been recognized by many studies [15,32,33,34,35]. Firstly, high levels of platelets might promote cytokine production, increase the blood supply of tumor cells, and protect tumor cells from damage [36]. Secondly, it might promote the activation of the coagulation mechanism, leading to tumor-related complications such as thrombus, thus increasing the risk of death.

Autoimmunity plays a dual role in tumor killing and cancer promotion [37]. As an important part of the immune system, lymphocytes played an important role in anti-tumor cell immunity in MPM patients [38,39] On the one hand, lymphocytes can directly kill tumor cells [40], promoting cytokine production [41], affecting the effect of immunotherapy [42], and preventing tumor micro-metastasis [43]. On the other hand, T lymphocyte subsets, including type II NK T lymphocytes [44], Treg cells [45], and B lymphocytes [46], can create an immunosuppressive microenvironment that inhibits anti-tumor immunity and promotes tumor growth, and ultimately promote tumor progression [37,47]. One of the main side effects of chemotherapy is cytopenia including lymphocytopenia [48]. This immunosuppression may be attenuated. This study found that a high level of lymphocytes was a favorable prognostic factor, which was consistent with previous studies [16,49]. It showed that in MPM, the balance of lymphocytes might be inclined to inhibit tumor progression.

The total protein can reflect the individual nutritional status and disease severity [50]. On the one hand, hypoproteinemia may indicate the patient’s older age and potential adverse performance status, including decreased food intake, decreased activity tolerance, and excessive consumption of the tumor on the body. Taken together, it may cause cachexia and ultimately affect the prognosis of patients [51]. On the other hand, the tumor microenvironment stimulates the immune system [23]. Total proteins, including albumin and globulin, can reflect the inflammatory state [52]. The results of this study show that patients with low total protein in serum may have a poor prognosis. At present, no studies have explored the correlation between the total protein in serum and the prognosis of MPM, but some studies have shown that a low total protein level in pleural effusion was a poor prognostic factor for MPM [53].

### 4.2. Pathological Features of Malignant Pleural Mesothelioma

On thoracoscopy, MPM initially appeared as small nodules covering the surface of the visceral and parietal pleura. As the disease progressed, the advanced stage showed diffuse patchy thickening formed by nodular fusion, which was not easy to clamp; the color might be yellow, dark red or black-gray. Different subtypes had different histological and immunohistochemical characteristics. The epithelial type was mainly cubic or flat cells, arranged into tubes, papillary, glandular, solid and other structures. In the sarcomatous type, fibroblast-like spindle cells were arranged in bundles or in a disorderly arrangement. The biphasic type was composed of epithelioid and sarcomatoid components each of which was more than 10% of the tumor and the transitional zone could be seen. These findings are similar to those of previous studies [11,54].

Immunohistochemical staining plays a very important role in the pathological diagnosis of MPM [55]. MPM is mainly distinguished from metastatic disease by calretinin, CK 5/6, WT-1 and D2-40 [11,56]. In addition, MPM is mainly distinguished from benign reactive mesothelial hyperplasia by BAP1 and MTAP [57,58]. In conclusion, a combination of at least two positive mesothelial (calretinin, CK 5/6, WT-1 and D2-40) and at least two negative adenocarcinoma immunohistochemical markers (TTF1, CEA and Ber-EP4) should be used in the differential diagnosis of MPM [59].

### 4.3. Radiological Characteristics of Malignant Pleural Mesothelioma

CT is the most commonly used tool to evaluate the disease status, stage and metastasis range of MPM patients [60]. This study found that most of the MPM tumors were unilateral, and the bilateral thoracic cavity was less involved [61]. However, most patients had malignant pleural effusion [59] and pleural thickening. The chest CT findings of malignant pleural mesothelioma patients were mostly oval, hump-shaped, nodular, wavy and circular pleural thickening, which existed simultaneously. The thickened pleura generally has obvious uneven enhancement. Cysts and necrosis may occur when large masses are formed. In addition, pleural effusion and mediastinal fixation are usually combined. The volume of the thoracic cavity on the affected side may also be reduced [62]. In this study, the incidences of parenchymal lung metastasis, rib metastasis, and chest wall involvement were low. This suggested that the patient staging in this study is low, which may potentially explain why the prognosis of patients in this study is relatively good. However, this study did not find any correlation between imaging features and histological subtypes, which may require further exploration in a large sample study in the future.

### 4.4. PET-CT Metabolic Features of Malignant Pleural Mesothelioma

Previous studies have shown that the overall diagnostic accuracy of PET-CT in pleural malignant tumors is 88%–95% sensitivity and 35%–100% specificity, respectively [59]. Compared with benign diseases, the uptake of tracer in pleural-affected areas of MPM patients was significantly higher [63,64]. It has been found that the SUVmax threshold of >2.0 can accurately distinguish malignant and benign pleural diseases with high sensitivity and specificity [65]. It is also helpful to differentiate extrathoracic diseases, especially lymph node involvement [66]. Previous studies have shown that there are significant differences in the OS and SUVmax between epithelioid subtype and non-epithelioid subtype MPM patients [67]. Sarcoma cases are the most aggressive; the SUVmax was usually the highest and the prognosis was the worst [68]. In addition, some studies suggest that PET-CT can also monitor the effect of radiotherapy, chemotherapy and immunotherapy in MPM patients [69,70,71].

### 4.5. Limitations

This study has some limitations. Firstly, although this was a two-center study, due to the rarity of the disease, the total included population was still relatively small. Secondly, due to the nature of the study, selection bias was inevitable. Thirdly, the time span of patients included in this study was relatively large, which might affect the results. Fourthly, due to the lack of relevant indicators of SUVmax, CRP and pleural effusion, we did not include the SUVmax, LENT score, PROMISE score and other indicators as independent variables in COX univariate analysis. Finally, the correlations between age, platelet and total protein and prognosis in this study were based on exploratory analysis (it did not reach the threshold after Bonferroni correction even though some were close). Therefore, these results should be replicated in the future.

## 5. Conclusions

In conclusion, as a rare and highly invasive malignant tumor, MPM has received increasing attention from clinicians. Through the combination of clinical, pathological, radiological and PET-CT metabolic results, the diagnostic accuracy of clinicians could be improved. According to the clinical laboratory indicators related to the identification of prognosis, including age, ESR, lymphocytes, platelets and total protein, the possible prognosis of patients could be inferred, which could prompt clinical experts to select more appropriate treatments for patients.

## Figures and Tables

**Table 1:** Baseline characteristics and laboratory variables of the study population.

Characteristic	Patients
Age (Median, IQR)	65.5 (56.0, 70.0)
Gender	
Male	71 (55.0)
Female	58 (45.0)
Smoke history	
Never	77 (59.7)
Current and former	52 (40.3)
Asbestos exposure	
No	102 (79.1)
Yes	27 (20.9)
ECOG PS	
0–1	107 (82.9)
2–3	22 (17.1)
Diagnostic methods	
Cell blocks from malignant pleural effusion	3 (2.3)
Percutaneous pleural needle biopsy	19 (14.7)
Video-Assisted Thoracic Surgery	12 (9.3)
Medical thoracoscopy	95 (73.6)
Histology	
Epithelioid	81 (62.8)
Non-epithelioid	48 (37.2)
Treatment	
Best supportive care	21 (16.3)
Chemotherapy ± anti-angiogenesis therapy	108 (83.7)
ESR (mm/h) (Median, IQR)	15.0 (8.0, 35.0)
Sodium(mmol/L) (Median, IQR)	141.0 (139.0, 142.3)
Potassium(mmol/L) (Median, IQR)	4.0 (3.7, 4.3)
Calcium(mmol/L) (Median, IQR)	2.2 (2.1, 2.3)
Chloride(mmol/L) (Median, IQR)	104.5 (102.7, 106.5)
WBC (10^9^/L) (Median, IQR)	6.5 (5.1, 8.3)
Neutrophil(10^9^/L) (Median, IQR)	4.2 (3.3, 5.8)
Lymphocyte(10^9^/L) (Median, IQR)	1.5 (1.2, 1.9)
Hemoglobin (g/L) (Median, IQR)	134.0 (120.5, 143.5)
Platelet(10^9^/L) (Median, IQR)	249.0 (194.0, 321.0)
Total protein(g/L) (Median, IQR)	63.9 (60.7,68.8)
Albumin(g/L) (Median, IQR)	36.8 (32.7, 39.7)
Glucose(mmol/L) (Median, IQR) Creatinine(umol/L) (Median, IQR)	5.0 (4.6,6.2)
Creatinine(umol/L) (Median, IQR)	61.2 (52.6,72.7)
Total bilirubin(umol/L) (Median, IQR)	10.6 (7.6, 14.4)

Abbreviations: SD, standard deviation; ECOG PS, Eastern Cooperative Oncology Group Performance Status; ESR, erythrocyte sedimentation rate; IQR, interquartile range; WBC, white blood cell.

**Table 2:** Univariable Cox regression analyses between baseline characteristics, laboratory variables and OS of malignant pleural mesothelioma patients.

Characteristic	No. (%)	HR	95%CI	*p* Value
Age (years)				
≤65.5	76 (58.9)	1		
>65.5	53 (41.1)	1.263	0.825–1.933	0.282
Gender				
Male	71 (55.0)	1		
Female	58 (45.0)	0.786	0.518–1.194	0.259
Smoke history				
Never	77 (59.7)	1		
Current and former	52 (40.3)	1.301	0.858–1.975	0.216
Asbestos exposure				
No	102 (79.1)	1		
Yes	27 (20.9)	0.979	0.599–1.599	0.931
ECOG PS				
0–1	107 (82.9)	1		
2–3	22 (17.1)	1.221	0.735–2.027	0.441
Diagnostic methods				
Cell blocks from malignant pleural effusion	3 (2.3)	1		0.249
Percutaneous pleural needle biopsy	19 (14.7)	2.540	0.334–19.306	0.368
Video-Assisted Thoracic Surgery	12 (9.3)	0.998	0.119–8.340	0.998
Medical thoracoscopy	95 (73.6)	1.875	0.259–13.582	0.534
Histology				
Epithelioid	81 (62.8)	1		
Non-epithelioid	48 (37.2)	1.450	0.955–2.202	0.081
Treatment				
Best supportive care	21 (16.3)	1		
Only Chemotherapy ± anti-angiogenesis therapy	108 (83.7)	0.478	0.269–0.848	0.012
ESR (mm/h)				
≤20.5	75 (58.1)	1		
>20.5	54 (41.9)	2.484	1.633–3.777	<0.001
Sodium (mmol/L)				
≤139.4	38 (29.5)	1		
>139.4	91 (70.5)	0.588	0.380–0.911	0.017
Potassium (mmol/L)				
≤4.5	117 (90.7)	1		
>4.5	12 (9.3)	2.044	1.104–3.785	0.023
Calcium (mmol/L)				
≤2.1	33 (25.6)	1		
>2.1	96 (74.4)	0.676	0.429–1.065	0.091
Chloride (mmol/L)				
≤102.9	35 (27.1)	1		
>102.9	94 (72.9)	0.656	0.421–1.023	0.063
WBC (10^9^/L)				
≤7.8	86 (66.7)	1		
>7.8	43 (33.3)	1.310	0.852–2.014	0.219
Neutrophil (10^9^/L)				
≤4.0	57 (44.2)	1		
>4.0	72 (55.8)	1.349	0.883–2.062	0.166
Lymphocyte (10^9^/L)				
≤1.1	26 (20.2)	1		
>1.1	103 (79.8)	0.531	0.329–0.857	0.009
Hemoglobin (g/L)				
≤124.5	40 (31.0)	1		
>124.5	89 (69.0)	0.664	0.430–1.025	0.065
Platelet (10^9^/L)				
≤289.5	90 (69.8)	1		
>289.5	39 (30.2)	1.807	1.166–2.801	0.008
Total protein (g/L)				
≤62.9	54 (41.9)	1		
>62.9	75 (58.1)	0.651	0.430–0.987	0.043
Albumin (g/L)				
≤37.5	75 (58.1)	1		
>37.5	54 (41.9)	0.654	0.421–1.018	0.060
Glucose (mmol/L)				
≤5.7	86 (66.7)	1		
>5.7	43 (33.3)	1.293	0.844–1.981	0.237
Creatinine (umol/L)				
≤65.8	77 (59.7)	1		
>65.8	52 (40.3)	1.290	0.853–1.951	0.228
Total bilirubin (umol/L)				
≤10.7	66 (51.2)	1		
>10.7	63 (48.8)	0.700	0.459–1.067	0.097

Abbreviations: HR, hazard ratio; CI, confidence interval; ECOG PS, Eastern Cooperative Oncology Group Performance Status; ESR, erythrocyte sedimentation rate; IQR, interquartile range; SD, standard deviation; WBC, white blood cell.

**Table 3:** Multivariable Cox regression analyses between baseline characteristics, laboratory variables and OS of malignant pleural mesothelioma patients.

Characteristic	HR	95%CI	*p* Value
Age (years)			
≤65.5	1		
>65.5	1.824	1.159–2.872	0.009
Gender			
Male	1		
Female	0.719	0.465–1.113	0.139
Histology			
Epithelioid	1		
Non-epithelioid	1.007	0.624–1.624	0.977
Treatment			
Best supportive care	1		
Chemotherapy ± anti-angiogenesis therapy	0.674	0.355–1.281	0.229
ESR (mm/h)			
≤20.5	1		
>20.5	2.197	1.318–3.664	0.003
Serum sodium (mmol/L)			
≤139.4	1		
>139.4	0.774	0.477–1.257	0.300
Serum potassium (mmol/L)			
≤4.5	1		
>4.5	1.397	0.710–2.747	0.333
Lymphocyte (10^9^/L)			
≤1.1	1		
>1.1	0.436	0.258–0.737	0.002
Platelet (10^9^/L)			
≤289.5	1		
>289.5	1.802	1.084–2.997	0.023
Total protein (g/L)			
≤62.9	1		
>62.9	0.625	0.394–0.990	0.045

Abbreviations: HR, hazard ratio; CI, confidence interval; ESR, erythrocyte sedimentation rate.

**Table 4:** IHC markers of the study population.

IHC Markers	Tested Patients	Patients *n* (%)	Epithelioid *n* (%)	Non-Epithelioid *n* (%)
Calretinin				
Negative	112	9 (8.0)	3 (4.2)	6 (14.6)
Positive		103 (92.0)	68 (95.8)	35 (85.4)
TTF-1				
Negative	107	105 (98.1)	65 (98.5)	40 (97.6)
Positive		2 (1.9)	1 (1.5)	1 (2.4)
CK5/6				
Negative	98	20 (22.5)	4 (7.1)	16 (48.5)
Positive		69 (77.5)	52 (92.9)	17 (51.5)
D2-40				
Negative	88	12 (13.6)	4 (7.4)	8 (23.5)
Positive		76 (86.4)	50 (92.6)	26 (76.5)
CEA				
Negative	84	73 (86.9)	48 (92.3)	25 (78.1)
Positive		11 (13.1)	4 (7.7)	7 (21.9)
Desmin				
Negative	81	67 (82.7)	41 (77.4)	26 (92.9)
Positive		14 (17.3)	12 (22.6)	2 (7.1)
MC				
Negative	81	15 (18.5)	4 (8.9)	11 (30.6)
Positive		66 (81.5)	41 (91.1)	25 (69.4)
Napsin-A				
Negative	72	71 (98.6)	49 (100.0)	22 (95.7)
Positive		1 (1.4)	0 (0)	1 (4.3)
WT-1				
Negative	68	8 (11.8)	3 (6.1)	5 (26.3)
Positive		60 (88.2)	46 (93.9)	14 (73.7)
CK7				
Negative	63	13 (20.6)	9 (27.3)	4 (13.3)
Positive		50 (79.4)	24 (72.7)	26 (86.7)
EMA				
Negative	60	10 (16.7)	3 (8.1)	7 (30.4)
Positive		50 (83.3)	34 (91.9)	16 (69.6)
CK				
Negative	58	1 (1.7)	1 (3.1)	0 (0)
Positive		57 (98.3)	31 (96.9)	26 (100.0)
Glut1				
Negative	51	7 (13.7)	7 (20.0)	0 (0)
Positive		44 (86.3)	28 (80.0)	16 (100.0)
Vimentin				
Negative	41	12 (29.3)	7 (33.3)	5 (25.0)
Positive		29 (70.7)	14 (66.7)	15 (75.0)
P53				
Negative	19	5 (26.3)	3 (25.0)	2 (28.6)
Positive		14 (73.7)	9 (75.0)	5 (71.4)

**Table 5:** Radiological characteristics of the study population.

Radiological Characteristics	Patients (*n* = 109) (%)	Epithelioid (*n* = 70) (%)	Non-Epithelioid (*n* = 39) (%)	*p* Value
Tumor location				0.376
Left	63 (57.8)	43 (61.4)	20 (51.3)	
Right	39 (35.8)	24 (34.3)	15 (38.5)	
Both	7 (6.4)	3 (24.3)	4 (10.2)	
Pleural invasion of interlobar fissure				0.185
No	73 (67.0)	50 (71.4)	23 (59.0)	
Yes	36 (33.0)	20 (28.6)	16 (41.0)	
Mediastinal pleural invasion				0.127
No	94 (86.2)	63 (90.0)	31 (79.5)	
Yes	15 (13.8)	7 (10.0)	8 (20.5)	
Mediastinal lymph node invasion				0.463
No	80 (73.4)	53 (75.7)	27 (69.2)	
Yes	29 (26.6)	17 (24.3)	12 (30.8)	
Thoracostenosis				0.108
No	92 (84.4)	62 (88.6)	30 (76.9)	
Yes	17 (15.6)	8 (11.4)	9 (23.1)	
Volume of pleural effusion				0.652
None	2 (1.8)	2 (2.9)	0 (0)	
Small	8 (7.4)	6 (8.6)	2 (5.1)	
Moderate	70 (64.2)	44 (62.9)	26 (66.7)	
Large	29 (26.6)	18 (25.6)	11 (28.2)	
Pleural thickening				0.342
None	3 (2.8)	1 (1.4)	2 (5.1)	
Regular	20 (18.3)	13 (18.6)	7 (17.9)	
Annular	9 (8.3)	6 (8.6)	3 (7.7)	
Lumpy	14 (12.8)	12 (17.1)	2 (5.1)	
Nodular	63 (57.8)	38 (60.3)	25 (64.2)	
Pleural calcification				0.054
No	87 (79.8)	52 (74.3)	35 (89.7)	
Yes	22 (20.2)	18 (25.7)	4 (10.3)	
Parenchymal lung metastasis				0.234
No	95 (87.2)	63 (90.0)	32 (82.1)	
Yes	14 (12.8)	7 (10.0)	7 (17.9)	
Rib metastasis				0.672
No	107 (98.2)	69 (98.6)	38 (97.4)	
Yes	2 (1.8)	1 (1.4)	1 (2.6)	
Chest wall involvement				0.344
No	94 (86.2)	62 (88.6)	32 (82.1)	
Yes	15 (13.8)	8 (11.4)	7 (17.9)	
Tumor necrosis				-
No	109 (100.0)	70 (100.0)	39 (100.0)	
Yes	0 (0)	0 (0)	0 (0)	
Pericardial effusion				-
No	109 (100.0)	70 (100.0)	39 (100.0)	
Yes	0 (0)	0 (0)	0 (0)	

## Data Availability

The datasets used and/or analyzed during the current study are available from the corresponding author on reasonable request.
